# Water hyacinth plant extract mediated green synthesis of Cr_2_O_3_/ZnO composite photocatalyst for the degradation of organic dye

**DOI:** 10.1016/j.heliyon.2021.e07652

**Published:** 2021-07-22

**Authors:** Osman Ahmed Zelekew, Paulos Asefa Fufa, Fedlu Kedir Sabir, Alemayehu Dubale Duma

**Affiliations:** a Department of Materials Science and Engineering, Adama Science and Technology University, Adama, Ethiopia; b Department of Applied Chemistry, Adama Science and Technology University, Adama, Ethiopia; c Nanotechnology Directorate, Ethiopian Biotechnology Institute, Ministry of Innovation and Technology, Addis Ababa, Ethiopia

**Keywords:** *Eichhornia crassipes*, Plant extract, ZnO, Cr_2_O_3_, MB dye, Visible light

## Abstract

The Cr_2_O_3_/ZnO composite catalysts with varying the amount of chromium precursors abbreviated as 0.02CrZn, 0.04CrZn, 0.06CrZn, 0.08CrZn, 0.1CrZn, and fixed the amount of Zn precursor (0.1 M) were prepared by using water hyacinth (*Eichhornia crassipes)* extract as a template/capping agent. The prepared catalysts were characterized and the catalytic performances of the catalysts were also checked for the degradation of methylene blue (MB) dye. The photocatalytic MB dye degradation by 0.08CrZn catalyst was achieved and 85% of MB dye was degraded within 90 min irradiation time. However, 0.1CrZ, 0.06CrZ, 0.04CrZ, 0.02CrZ, ZnO, and Cr_2_O_3_ catalysts degrade only 80, 74, 79, 76, 52, and 74% of MB dye, respectively. The catalytic performances indicated that the addition of optimum amount of chromium precursor in the preparation of Cr_2_O_3_/ZnO composite catalysts with the aid of *Eichhornia crassipes* plant extract enhances the catalytic activities. This performance enhancement could be as a result of reducing the electron/hole pair separation and the porosity resulted from the plant extract in the catalyst system.

## 1 Introduction

Recently, the fast-growing industries and population size lead to the formation of water pollution which causes the environment and human health problems [1, 2, 3]. The wastes from industrial area such as textile dyes, pharmaceutical, dyeing, paper and pulp industries are the main sources of organic and inorganic compounds and a cause for water pollution [4]. Particularly, organic dyes released from industries had severe health problems, toxic, indestructible, bio-recalcitrant, mutagenic, carcinogenic and have potential to fade resistant [5, 6]. Due to this reason, appropriate methods are needed to remove these organic pollutants before they are released to the environment [7, 8, 9]. Among the methods, photocatalysis is one of effective and efficient candidate technology used in wastewater treatment [10, 11, 12, 13]. The method is low cost, environmental benign and leads to complete mineralization of organic pollutants from wastewater [14, 15]. Among the promised photocatalys, semiconductor based heterogeneous catalysts have been studied and reported as efficient and effectively treatments methods [13, 14, 16, 17] Semiconductor photocatalysts materials such as zinc oxide (ZnO), titanium dioxide (TiO_2_), iron oxide (Fe_2_O_3_), and tungsten trioxide (WO_3_) have been widely studied for the wastewater treatment [18, 19, 20, 21, 22]. ZnO, in particular, is considered as candidate materials for the degradation organic pollutants owing to its chemical stability, low cost, non-toxicity, and high photosensitivity [23, 24]. However, ZnO exhibits large band gap energy (3.2 eV) which could have limited application due to high electron-hole pairs recombination rate [25, 26]. Hence, combination of ZnO with other semiconductors, to enhance the catalytic efficiency, is highly needed.

There are many reports on ZnO based photocatalysts for the removal of pollutants [27, 28, 29]. Different methods such as doping, making p-n heterojunctions with ZnO have been widely studied to overcome the limitations of ZnO semiconductor material [30]. Particularly, the synthesis of p-n heterojunctions structures was introduced as the best method to solve the fast recombination rates of electron-hole pairs [31]. This is due to the formation of built-in potential across the junction which causes as the electrons and holes generated moves in opposite to improve the lifetime of charge carriers [32]. Among the *p*-type semiconductor materials, Cr_2_O_3_ owing its smaller band gap energy of about 2.3 eV [33], was selected due to its high chemical and thermal stability [34], and its huge applications in catalysis [33, 35]. Many researchers have also reported the ability of Cr_2_O_3_ to degrade organic compounds under visible-light [36, 37]. For this reason, ZnO/Cr_2_O_3_ nanocomposite photocatalyst was used in this particular work to improve the photocatalytic efficiency of ZnO photocatalyst.

However, ZnO combinations with other lower band gap semiconductors materials or doping with metal and non-metals may not be enough to overcome the lower photocatalytic activity. Hence, porous metal oxides based photocatalysts preparation with combining of lower band gap semiconductors materials could have also many advantages on the wastewater treatment [38, 39, 40, 41]. For this reason, photocatalysts synthesis by using biological renewable sources as a template is green and environmentally friendly for treatments of wastewater [42, 43, 44, 45, 46, 47]. Among the natural biological renewable resources, *Eichhornia crassipes* is one of unwanted and weeds plant that could give a potential application and used as a template for the synthesis of photocatalysts in wastewater treatment.

In this paper, the water hyacinth extract was used in the synthesis Cr_2_O_3_/ZnO composite catalysts. The Cr_2_O_3_/ZnO photocatalysts were characterized by different instruments and tested towards the degradation of MB under visible light. It is expected that the Cr_2_O_3_/ZnO composite photocatalysts could have enhanced photocatalytic activities as a result of widening the absorption range of light, reduced electron-hole recombination rates, and enhancing the porosity of the catalysts due to water hyacinth plant extract during the preparation of the catalysts.

## 2 Materials and methods

### 2.1 Chemicals and reagent

Chromium nitrate (Cr(NO_3_)_2_.H_2_O, sodium hydroxide (NaOH), zinc nitrate hexahydrate (Zn(NO_3_)_2_.6H_2_O), and ethanol were used in this experiment. The reagents and chemicals were used without any further purification.

### 2.2 Preparation of *Eichhornia crassipes* extracts

The water hyacinth plants samples were collected from Lake Koka, Ethiopia and dried at room temperature. Then, the dried sample was crushed and 15 g of crushed plant powder was added into distilled water (400 mL) and stirred at 50 °C for 1 h. Then, the resulting solution was filtered and the plant extract was kept for further application.

### 2.3 Green synthesis of *Eichorinia crassipes* plant mediated ZnO/Cr_2_O_3_ composite photocatalyst

The plant extract mediated ZnO/Cr_2_O_3_ catalyst was synthesized with facile method. In this experiment, a certain amount of Zn(NO_3_)_2_. H_2_O solution was added drop by drop in to a beaker containing 20 mL of plant extract. The resulting solution was then heated at 50 °C with constant stirring. A certain amount of Cr(NO_3_)_2_.H_2_O solution was added drop-wise to the solution after 30 min and the pH was adjusted to 10 after 1 h. Then, the precipitated result was washed with water and ethanol. Finally, the sample was dried in oven at 60 °C for 24 h and calcined at 500 °C for 2 h. With the molar ratio of Cr/Zn, 0.1/0.1, 0.08/0.1, 0.06/0.1, 0.04/0.1, 0.02/0.1, and ZnO only in the presence of plant extract were synthesized and abbreviated as 0.1CrZ, 0.08 CrZ, 0.06CrZ, 0.04 CrZ, 0.02 CrZ, and Z, respectively.

### 2.4 Characterizations

The Shimadzu XRD- 7000 was used for X-ray diffraction (XRD) analysis. The morphology of the sample was checked by field-emission scanning electron microscopy (FESEM, JSM 6500F, JEOL). The 65 FT-IR (PerkinElmer) spectroscopy was used for Fourier transform infrared (FTIR) analysis. The MB dye degradation was evaluated by Shimadzu–3600 Plus UV–vis spectrophotometer.

### 2.5 Catalytic activity measurements

The degradation of MB was performed under visible light conditions as follows. Specifically, 10 ppm of MB (125 mL) aqueous solution was added into the reactor glass. Next, 25 mg of composite catalyst was added into the reactor containing an aqueous solution of the MB dye and stirred for 30 min for adsorption and desorption equilibrium purpose under dark condition. After 30 min, the visible light source with halogen lamp (Japan, 150 W) was turned on and water was circulated for cooling purpose. Then, the aliquot (5 mL) was withdrawn at different time interval and analyzed with UV-vis spectroscopy. The concentrations of the MB left were checked by using maximum absorbance value of MB at wavelength 663 nm (*λ*_max_ = 663 nm).

Moreover, the reusability of the catalyst was also tested. In details, 75 mg of the 0.08ZCr catalyst was added in to 10 ppm of MB (375 mL) and the mixture was stirred for 30 min. Subsequently, the resulting mixture was exposed to the visible light for 90 min. Subsequently, the aliquot solution (5 mL) was taken out from the mixture. The catalyst was separated by washing using centrifuge and used for the next cycle.

## 3 Results and discussion

**Figure 1:**
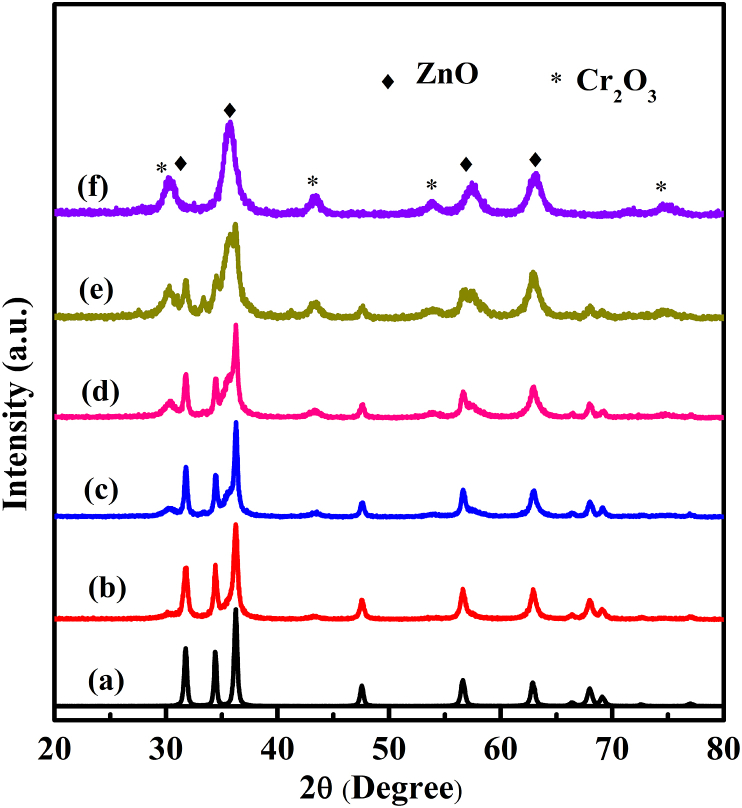
XRD patterns for (a) ZnO(Z), (b) 0.02CrZ, (c) 0.04 CrZ, (d) 0.06 Cr Z, (e) 0.08CrZ, and (f) 0.1CrZ.

The Cr_2_O_3_/ZnO was synthesized using water hyacinth extract as a template/capping agent for the degradation of organic dye application. The prepared powder catalysts phases and structure was checked by XRD. Figure 1(a-f) indicates the XRD for ZnO, and ZnO/Cr_2_O_3_ synthesized in the presence of *Eichhornia crassipes* extract with adding different amount of chromium precursor. The (100), (002), (101), (102), (110), (103), (200), (112) and (201) planes shows hexagonal wurtzite ZnO with corresponding major diffraction peaks at 31.76°, 34.43°, 36.25°, 47.54°, 56.59°, 62.86°, 66.37°, 67.95° and 69.08°, respectively (JCPDS No. 36–1451). Moreover, the XRD peaks of Cr_2_O_3_ with (104), (110), and (116) planes at 33.61°, 36.30°, and 54.98° major peaks, respectively, was also observed (JCPDS No. 038-1479). As it is observed from the XRD Figure, increasing the amount of chromium precursor with 0.02, 0.04, 0.06, 0.08 and 0.1 M and keeping the concentration of Zn precursor constant (0.1 M), illustrates that the peaks for Cr_2_O_3_ is shown clearly. The results indicated that the Cr_2_O_3_/ZnO composite catalyst was synthesized successfully without any other impurity phases.

**Figure 2:**
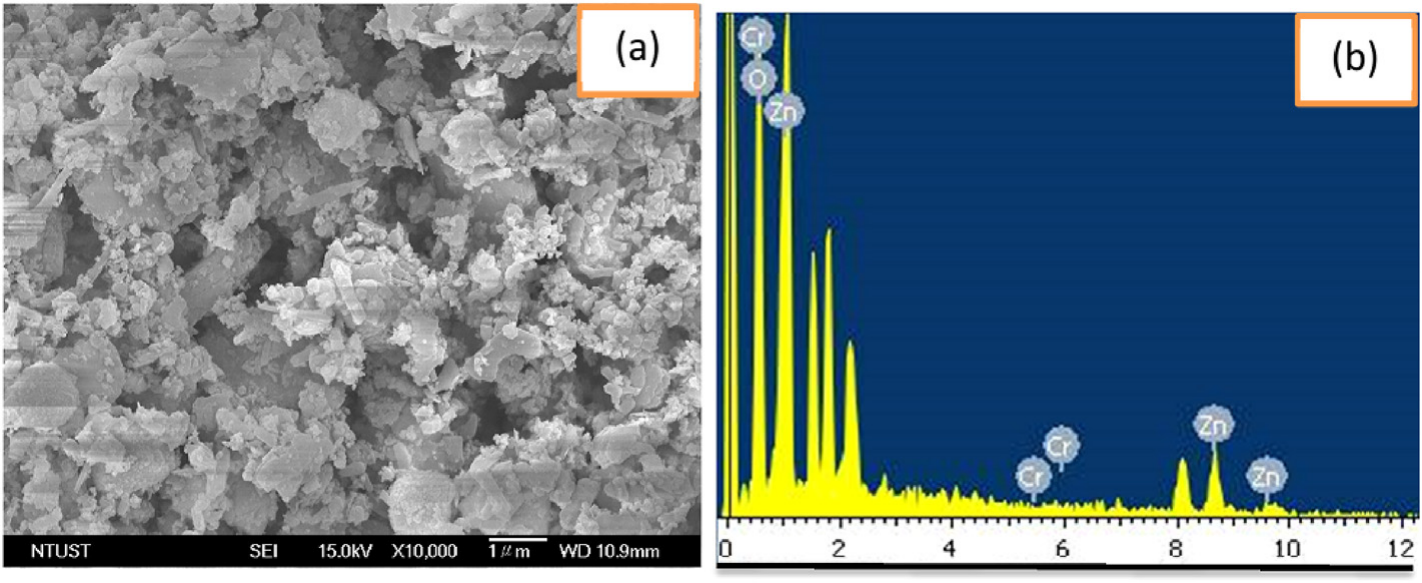
(a) SEM image and (b) EDS for 0.08CrZ sample synthesized with *Eichhornia crassipes* plant extract.

The SEM analysis was also used to check the surface morphologies of the synthesized sample. Figure 2 (a-b) shows the SEM image and EDS of the 0.08CrZ sample. The aggregated surface morphologies with sheet were shown in the Figure 2a. Moreover, Figure 2b indicates the EDS of the 0.08CrZ sample. The Zn, Cr, and O elements from Figure 2b were clearly shown. It can be concluded that Cr_2_O_3_/ZnO composite catalyst was synthesized successfully.

**Figure 3:**
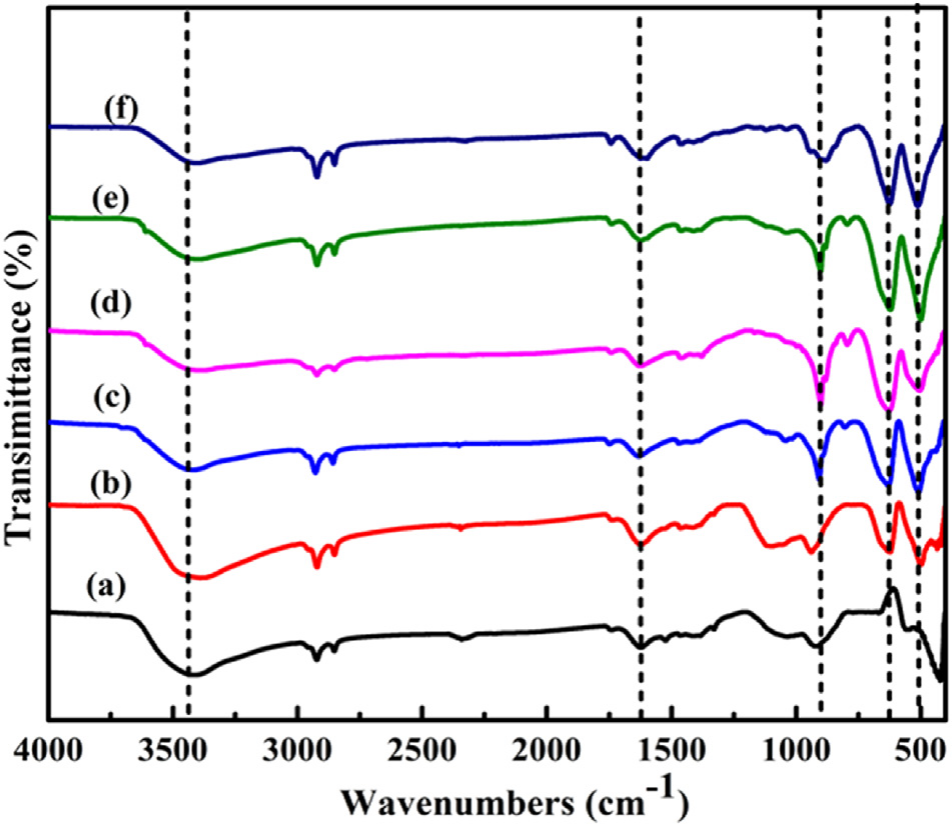
FT-IR spectra of (a) ZnO, (b) 0.02CrZ, (c) 0.04 CrZ, (d) 0.06 Cr Z, (e) 0.08CrZ, and (f) 0.1CrZ.

The FTIR spectra of the samples are also shown in Figure 3. The FTIR band characteristics assigned at 1650 cm^−1^ and 3450 cm^−1^ corresponds to bending modes of adsorbed water molecules and the OH stretching of modes of water molecules, respectively [48, 49]. The Cr-O vibration band is also assigned at 511 cm^−1^ [50]. Moreover, the band around and 623 cm^−1^ may be also attributed to the Cr-O binding mode [48, 51]. Stretching vibration modes of Zn-O is located at around 420 cm^−1^ characteristic peaks as reported in literature [52, 53]. Hence, it can be conclude that the Zn-O and Cr-O bond were formed successfully which are also the indicators for metals and oxygen bond formations in the composite catalysts.

**Figure 4:**
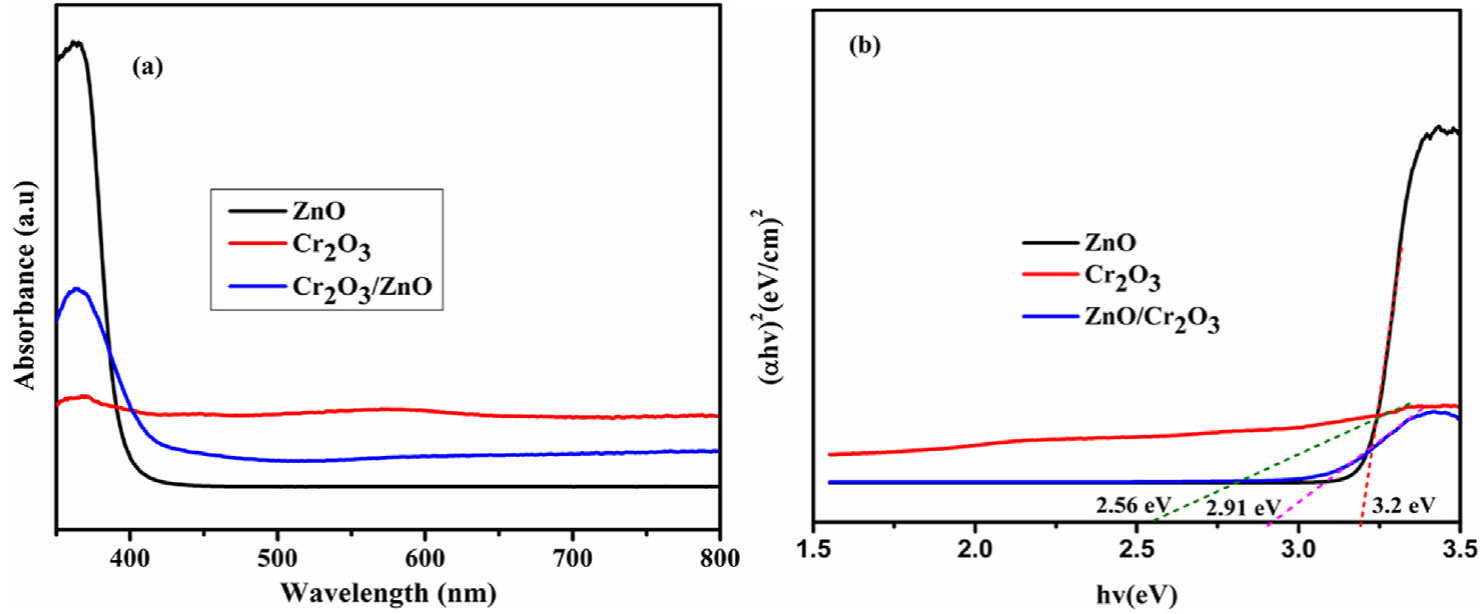
(a) Ultraviolet-visible absorption spectrum and (b) the (α*hv*)^2^-*hv* plot from the ultraviolet-visible absorption spectrum for Cr_2_O_3_, ZnO, and 0.08CrZn samples.

The ultraviolet-visible absorption spectrum and the (α*hv*)^2^-*hv* plot from the ultraviolet-visible absorption spectrum for Cr_2_O_3_, ZnO, and 0.08CrZn samples are indicated in Figure 4a-b. The pure ZnO illustrates broad adsorption in UV-region due to its wide band gap while the Cr_2_O_3_ absorption was in visible region. The optical absorption edges and absorption intensity of ZnO/Cr_2_O_3_ composite is enhanced. It might be attributed to the presence of Cr_2_O_3_ in the ZnO sample (Figure 4a). The classic Tauc approach was used to calculate bandgap of the samples according to Eq. (1) as shown below [54, 55].

(1) $${\left(\alpha hv\right)}^{\frac{1}{n}}=k\left(hv-E_{g}\right)$$

Where α is the absorbance coefficient, *h* the Planck constant, *k* the absorption constant for a direct transition, *hν* the absorption energy, and *E*_g_ the band gap. As shown in Figure 4b, the band gap energies for ZnO, Cr_2_O_3_, and ZnO/Cr_2_O_3_ (0.08CrZn) were 3.2, 2.56, and 2.91 eV, respectively.

**Figure 5:**
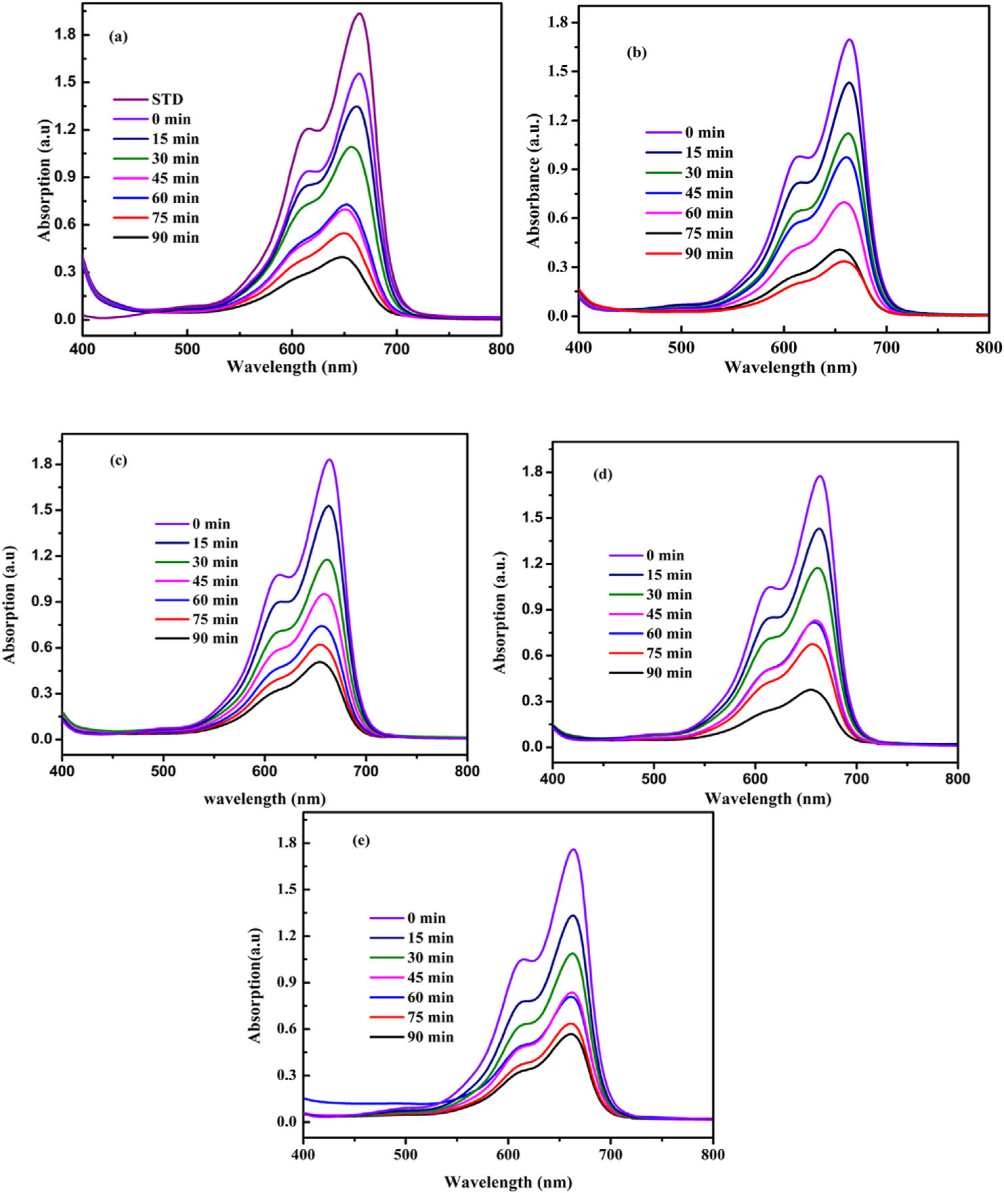
UV-Visible absorption spectra for (a) 0.1CrZ (b) 0.08CrZ, (c) 0.06CrZ, (d) 0.04CrZ, and (e) 0.02 CrZ catalyst after different time irradiations.

To check the photocatalytic activities of the composites photocatalysts, MB was selected as the target organic pollutants. Figure 5 illustrates the UV-visible absorption spectra for the catalytic activities of ZnO and ZnO/Cr_2_O_3_ prepared with different amount of Cr precursor in the presence of plant extract. As it is observed from the Figure 5a-e, the degradation was performed under visible light within 90 min irradiation in the interval of 15 min.

**Figure 6:**
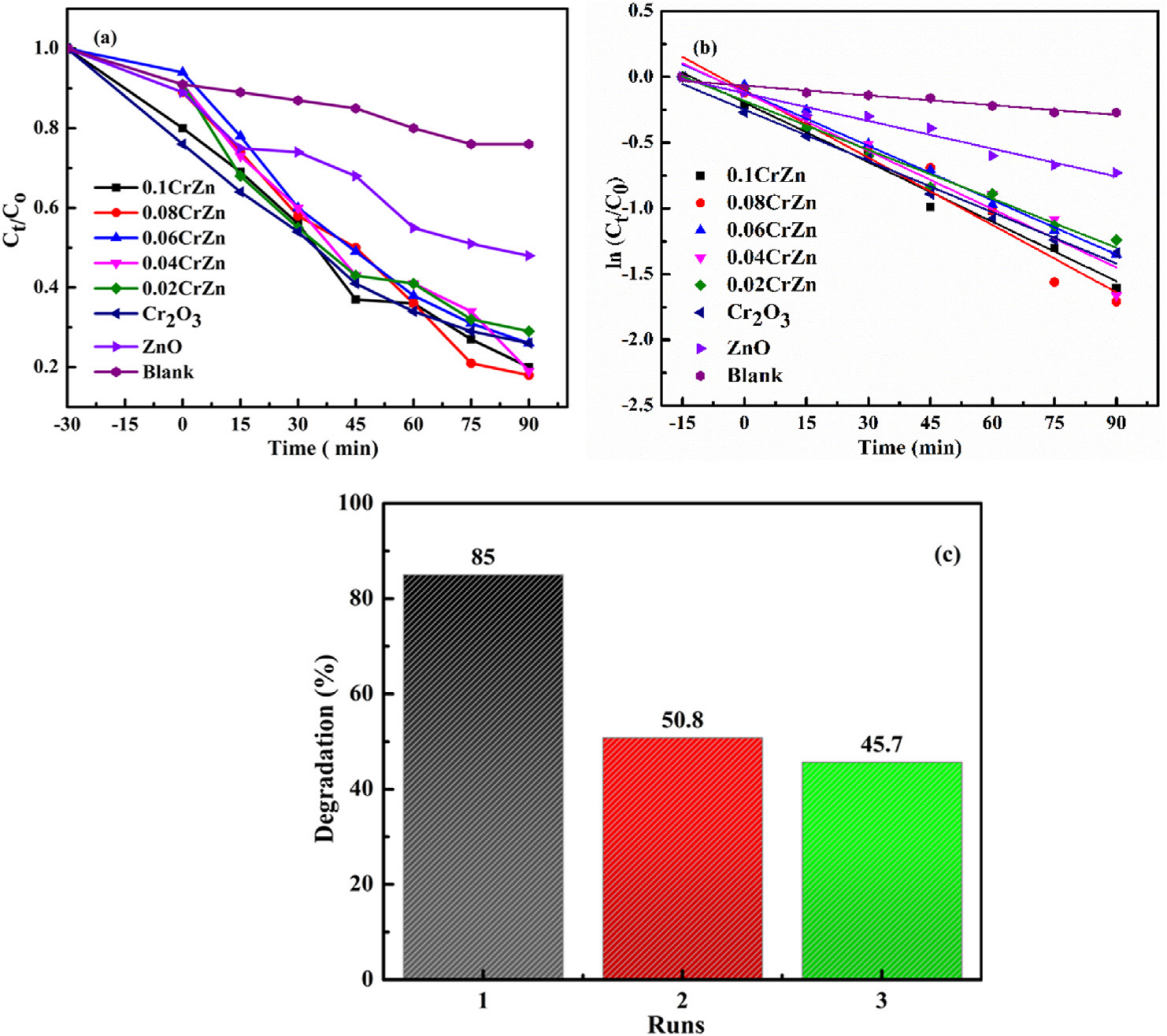
(a) C_t_/C_0_ plots, (b) the first-order kinetic plot at different irradiation times for blank, 0.1CrZ, 0.08 CrZ, 0.06CrZ, 0.04CrZ, 0.02CrZ, ZnO, and Cr, and (c) the stability test for 0.08 CrZ sample. (c) The reusability efficiency for 0.08 CrZ catalyst.

Moreover, Figure 6(a) indicates C_t_/C_o_ ratio of the first-order kinetic plot for different catalysts. As it is observed from Figure 6(a), in the presence of the catalyst 0.08CrZn, 85% of degradation was accomplished within 90 min irradiation time. However, 0.1CrZ, 0.06CrZ, 0.04CrZ, 0.02Cr, ZnO, and Cr_2_O_3_ catalysts degrade only 80, 74, 79, 76, 52, and 74% of MB dye, respectively. The results indicated that the optimum amount of Cr precursor addition on Zn precursor in the presence of water hyacinth plant extract showed enhanced photocatalytic activity. The enhanced photocatalytic activity may be as results of the electron/hole pairs separation and the porosity of the material resulted from water hyacinth plant extracts.

The kinetic rate removal of MB dye was illustrated according to the following Eq. (2) shown below [32, 56].

(2) $$\text{ln}\left(\frac{C_{t}}{C_{0}}\right)=-\text{kt}$$

Where: *C*_0_ and *C*_t_ are the initial concentration and the concentration at a time (t), respectively, and k is the rate constant. Figure 6b shows the plot of the rate constants for ZnO, Cr_2_O_3_, and Cr_2_O_3_/ZnO with varying the amount of Cr precursor in the presence of water hyacinth plant extract. As shown from the Figure 6b, the MB degradation indicates the pseudo-first-order kinetics [32]. The estimated rate constants of 0.1CrZ, 0.08 CrZ, 0.06CrZ, 0.04CrZ, 0.02CrZ, ZnO, and Cr_2_O_3_ were 0.015, 0.017, 0.014, 0.015, 0.012, 0.013, 0.002, and 0.007 min^−1^, respectively. The result shows the highest rate constant was obtained from 0.08 ZCr catalysts. The photo-stability of 0.08 ZCr was also investigated under repeated experiments. As shown in Figure 6c, the catalyst still works after three repeated experiments.

**Figure 7:**
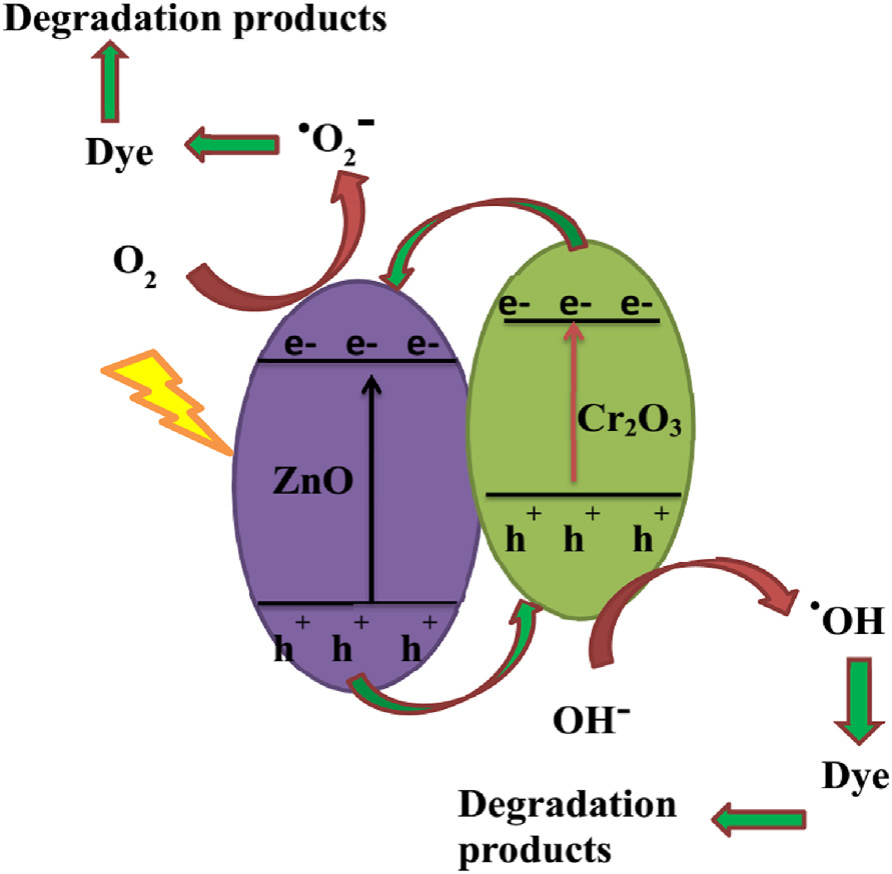
Proposed degradation mechanism of organic dye with Cr_2_O_3_/ZnO composite photocatalyst under light irradiation.

The degradation of organic dye mechanism is also showed in Figure 7. The catalytic activities of Cr_2_O_3_/ZnO was improved due to separation of electrons/holes pair, and improving visible light absorption range [57]. Moreover, the formation of p–n heterojunction is also responsible for the electron and hole separation in which the catalytic activities of the materials will be increased. In the photocatalysis reaction, the electrons will be moved to the n-type ZnO conduction band and the holes will be moved to p-type Cr_2_O_3_ valence band. The electrons migrated in the conduction band of ZnO will be interacted with oxygen and superoxide radical anions (O_2_^-^) will be generated. Moreover, the holes in the valence band of the p-type Cr_2_O_3_ will be reacted with OH^−^ and H_2_O and the reactive oxygen species (^**.**^OH) will be formed. Then, **·**OH and **·**O_2_^-^ could be reacted with MB dye and decomposition of the organic dye in to H_2_O and CO_2_ is expected [58, 59]. Therefore, Cr_2_O_3_/ZnO composite catalytic efficiency could be improved due to lowering the electron-hole pair recombination rates resulted from p–n heterojunction formation and the catalyst porosity due to the presence of *Eichhornia crassipes* plant extract in the preparation.

## 4 Conclusion

The green method preparation of Cr_2_O_3_/ZnO composite photocatalyst with the aid of water hyacinth extract as a template/capping agent was used. The catalytic degradation activities of the Cr_2_O_3_/ZnO composite photocatalysts were tested toward MB dye and 0.08CrZn catalyst was achieved with 85% degradation efficiency. However, 0.1CrZ, 0.06CrZ, 0.04CrZ, 0.02Cr, ZnO, and Cr_2_O_3_ catalysts, degrades only 80, 74, 79, 76, 52, and 74% of MB dye, respectively. The addition of optimum amount of Cr in the preparation of Cr_2_O_3_/ZnO catalysts with water hyacinth plant extract enhances the catalytic activities. The photocatalytic enhancement could be due to the electron/hole separations as a result of p–n heterojunction formation and the porosity of the catalyst resulted from water hyacinth extract. Therefore, the water hyacinth extract mediated Cr_2_O_3_/ZnO composite catalyst could be used in the treatments of polluted water.

## Declarations

### Author contribution statement

Osman Ahmed Zelekew: Conceived and designed the experiments; Analyzed and interpreted the data; Wrote the paper.

Paulos Asefa Fufa: Performed the experiments; Wrote the paper.

Fedlu Kedir Sabir: Analyzed and interpreted the data.

Alemayehu Dubale Duma: Analyzed and interpreted the data; Wrote the paper.

### Funding statement

This work was supported by 10.13039/501100015758Adama Science and Technology University (10.13039/501100015758ASTU) (grant No. ASTU/AS-R/001/2019).

### Data availability statement

Data included in article/supplementary material/referenced in article.

### Declaration of interests statement

The authors declare no conflict of interest.

### Additional information

No additional information is available for this paper.
